# Nifedipine protects against overproduction of superoxide anion by monocytes from patients with systemic sclerosis

**DOI:** 10.1186/ar1457

**Published:** 2004-11-16

**Authors:** Yannick Allanore, Didier Borderie, Axel Périanin, Hervé Lemaréchal, Ohvanesse Garabed Ekindjian, André Kahan

**Affiliations:** 1 Rheumatology A Department, Paris V University, Assistance Publique-Hôpitaux de Paris, Cochin Hospital, Paris, France; 2 Biochemistry A Department, Paris V University, Assistance Publique-Hôpitaux de Paris, Cochin Hospital, Paris, France; 3 Cell Biology Department, Cochin Institute, CNRS UMR 8104, INSERM 0567, Paris, France

**Keywords:** monocyte, nifedipine, protein kinase C, superoxide anion, systemic sclerosis

## Abstract

We have reported previously that dihydropyridine-type calcium-channel antagonists (DTCCA) such as nifedipine decrease plasma markers of oxidative stress damage in systemic sclerosis (SSc). To clarify the cellular basis of these beneficial effects, we investigated the effects *in vivo *and *in vitro *of nifedipine on superoxide anion (O_2_^•-^) production by peripheral blood monocytes. We compared 10 healthy controls with 12 patients with SSc, first after interruption of treatment with DTCCA and second after 2 weeks of treatment with nifedipine (60 mg/day). O_2_^•- ^production by monocytes stimulated with phorbol myristate acetate (PMA) was quantified by the cytochrome *c *reduction method. We also investigated the effects *in vitro *of DTCCA on O_2_^•- ^production and protein phosphorylation in healthy monocytes and on protein kinase C (PKC) activity using recombinant PKC. After DTCCA had been washed out, monocytes from patients with SSc produced more O_2_^•- ^than those from controls. Nifedipine treatment considerably decreased O_2_^•- ^production by PMA-stimulated monocytes. Treatment of healthy monocytes with nifedipine *in vitro *inhibited PMA-induced O_2_^•- ^production and protein phosphorylation in a dose-dependent manner. Finally, nifedipine strongly inhibited the activity of recombinant PKC *in vitro*. Thus, the oxidative stress damage observed in SSc is consistent with O_2_^•- ^overproduction by primed monocytes. This was decreased by nifedipine treatment both *in vivo *and *in vitro*. This beneficial property of nifedipine seems to be mediated by its cellular action and by the inhibition of PKC activity. This supports the hypothesis that this drug could be useful for the treatment of diseases associated with oxidative stress.

## Introduction

Systemic sclerosis (SSc) is a connective tissue disease, characterised by vascular involvement with generalised microangiopathy culminating in systemic fibrosis. Several lines of evidence suggest that the generation of oxygen free radicals is of major importance in the pathogenesis of SSc [1]. Frequent episodes of reperfusion injury generate oxygen free radicals locally, but increased lipid peroxidation is not related to Raynaud's phenomenon only [2] and the inflammatory process might also generate oxidative stress [1].

Histological studies of cutaneous SSc lesions have revealed early mononuclear cell infiltration of perivascular spaces around small vessels [3,4], and mononuclear cells might affect vascular and tissue lesions by producing various molecules [5]. Monocytes seem to have a key function in several disorders (for example atherosclerosis) associated with free radical generation [6]. Monocytes from patients with SSc produce greater amounts of superoxide anion (O_2_^•-^) than those from healthy subjects and patients with primary Raynaud's phenomenon [7].

Dihydropyridine-type calcium-channel antagonists (DTCCA) are an essential treatment in SSc because they decrease vasospastic propensity. These drugs are suspected to have anti-oxidant properties in other diseases [8,9]. We reported previously that nifedipine and nicardipine decrease circulating markers of oxidative stress damage in patients with SSc [10,11]. The aim of the present study was to investigate O_2_^•- ^production *ex vivo *by monocytes from patients with SSc after a wash-out and during nifedipine treatment. We also investigated the effects of nifedipine *in vitro *on O_2_^•- ^release from human monocytes, on protein phosphorylation with phorbol myristate acetate (PMA) as stimulator and on protein kinase C (PKC) activity.

## Methods

Reagents, except when specified, were provided by Sigma (St Louis, MO, USA).

### Patients

Twelve non-smoking patients with SSc were included (three men and nine women); their mean age was 56 (±10 SD) years and the mean disease duration was 8 ± 5 years. Each of the patients had been hospitalised for systematic follow-up of the disease. SSc was classified as limited cutaneous or diffuse cutaneous according to the criteria of LeRoy and colleagues [12]. The clinical features of their disease were assessed as recommended [13]; results are detailed in Table 1.

The initial evaluation included biological tests performed on the morning of admission, after 1 hour of rest at room temperature; patients stopped taking calcium-channel blockers 3 days before admission [10]. This wash-out period was long enough for DTCCAs to have ceased to have an effect because the half-lives of nifedipine and nicardipine lie between 6 and 11 h and these drugs are converted into inactive metabolites. Patients were also evaluated 2 weeks later, when they were receiving stable treatment (including 20 mg of nifedipine three times a day). As before, this was done in the morning, after 1 h of rest at room temperature. All patients gave written informed consent. The control subjects were 10 healthy non-smokers from the laboratory staff (8 women and 2 men, mean age 48 ± 9 years).

### Monocyte preparation

Blood samples (20 ml) were collected on EDTA. Whole blood was diluted 1:2 in RPMI 1640 solution (Eurobio, Les Ulis, France), layered on 15 ml of Ficoll-Hypaque (relative density 1.077; Eurobio) and centrifuged at 150 *g *for 25 min at room temperature to separate mononuclear cells from red blood cells and polymorphonuclear neutrophils. Mononuclear cells collected at the interface were washed twice in RPMI 1640 solution and separated into lymphocytes and monocytes by gradient centrifugation with 47% Percoll (Pharmacia, Uppsala, Sweden) at 150 *g *for 25 min. The purity of the monocyte population (more than 90%) was assessed by May–Grunwald–Giemsa staining. Cells were diluted in RPMI and used immediately.

### O_2_^•- ^(respiratory burst) assay

O_2_^•- ^was quantified by the cytochrome *c *reduction assay at 550 nm [14] with a Uvikon^® ^spectrophotometer equipped with a thermostat-controlled cuvette holder and a magnetic stirrer. In brief, 840 µl of monocytes (2 × 10^6 ^cells) were incubated at 37°C with 80 µM cytochome *c *in 1 ml of PBS with stirring before stimulation with 10 µl of PMA (100 nM). Absorbance was read against a reference blank curve containing PBS and cytochrome *c *at 5, 10 and 15 min. Results are expressed in nmoles of O_2_^•- ^per 10^6 ^cells using 21.2 mM/cm as the extinction coefficient. For *in vitro *evaluation of the effects of DTCCAs, control monocytes were preincubated for 30 min with the desired concentration of drugs or vehicle (dimethyl sulphoxide, 100 µM) before stimulation with PMA.

The respiratory burst of control and nifedipine-treated monocytes induced by formyl-Met-Leu-Phe (fMLP) was measured by a sensitive fluorimetric assay for hydrogen peroxide under standard conditions [15] using the Amplex Red fluorescent probe (Molecular Probes, Eugene, OR, USA). The increase in the fluorescent signal (excitation and emission wavelengths of 530 and 590 nm respectively) was monitored continuously after the stimulation of monocytes with 1 µM fMLP. Results are expressed as the percentage of control values, representing the total hydrogen peroxide production.

The scavenging effect of nifedipine was studied in an assay system containing nifedipine at the desired concentration with 0.15 mM hypoxanthine, 1.5 mM EDTA and 0.025 mM cytochrome *c*. The reaction was started by adding 0.1 U/ml xanthine oxidase. Absorbance was read at 550 nm.

### Protein phosphorylation in monocytes and in a cell-free system

Phosphorylated proteins were detected by using the fluorescent Pro-Q-Diamond dye (Molecular Probes), which can directly detect phosphate groups attached to tyrosine, serine or threonine residues in gels. In brief, monocytes from healthy donors were treated with or without nifedipine and stimulated for 10 min with 100 nM PMA (which induces the activation of PKC). Reactions were stopped with ice-cold buffer and cell lysates were prepared by incubating monocytes for 30 min in 0.5 ml of ice-cold extraction buffer (350 nM NaCl, 20 mM Hepes-KOH, pH 7.9, 1 mM EDTA, 0.1 mM EGTA, 20% glycerol, 1% Nonidet P40, 1 mM dithiothreitol, 0.5 mM phenylmethylsulphonyl fluoride, 20 mM ß-glycerophosphate, 0.1 mM Na_3_VO_4 _and 1 µg/ml each of aprotinin, pepstatin and leupeptin). Cells were then disrupted by sonication (three times 5 s) and proteins were delipidated and desalted before being subjected to SDS gel electrophoresis. Protein phosphorylation was also studied after treatment of a cytosolic fraction of resting monocytes with PMA for 10 min. Protein samples (150 µg of a 1 mg/ml protein sample) were treated *in vitro *with nifedipine for 30 min before stimulation of PKC with a mixture of diacylglycerol and calcium. This was done with the PKC Biotrak^® ^enzyme assay (Amersham Biosciences, Little Chalfont, Buckinghamshire, UK) in accordance with the manufacturer's instructions. Proteins (50 µg) were separated by SDS gel electrophoresis. Proteins were fluorescently stained by fixing the gels overnight in 50% methanol and 10% trichloracetic acid. The gels were washed with deionised water for 10–20 min, stained with Pro-Q-Diamond for 180 min and destained by three washes in 4% acetonitrile in 50 mM sodium acetate, pH 4, for 2 hours. Gels were scanned with a fluorimager, a Typhoon 9400 laser scanner (Amersham Biosciences), with excitation at 532 nm and a 580 nm band pass emission filter for Pro-Q-diamond dye detection. Phosphorylated proteins were quantified densitometrically with the Image Quant software. Results represent the net phosphorylation induced by PMA, expressed as percentages of the control PMA lane (taken as 100%).

### PKC assay

PKC assays were performed *in vitro *with a biologically active full-length recombinant isoform (Calbiochem, EMB Biosciences, San Diego, CA, USA) and a non-radioactive PKC kinase assay kit (Stressgen, Victoria, BC, Canada). The principle is based on an enzyme-linked immunosorbent assay method that uses a specific synthetic peptide as a substrate for PKC and a polyclonal antibody that recognised the phosphorylated form of the substrate. In brief, 1 µl of recombinant PKC (5 µg/ml) in 30 µl of kinase assay dilution buffer was incubated for 20 min at 37°C with the drug vehicle (control) or with nifedipine or two other PKC inhibitors (calphostin and GFI09203). PKC was then activated by 10 µl of PMA (100 nM) for 10 min at 37°C and the reaction was blocked by incubating tubes in ice-cold water. PKC activity was then determined as recommended by the manufacturer. Results are expressed as percentages of the net activity induced by PMA (taken as 100%).

### Approval and consent

The study was approved by local ethics committee, and all patients gave written informed consent.

### Statistical analysis

Data were compared with the nonparametric Mann–Whitney (unpaired data) and Wilcoxon (paired data) tests. *P *< 0.05 was considered significant. All quantitative data are expressed as means ± SD.

## Results

### Monocyte O_2_^•- ^production and respiratory burst

**Figure 1:**
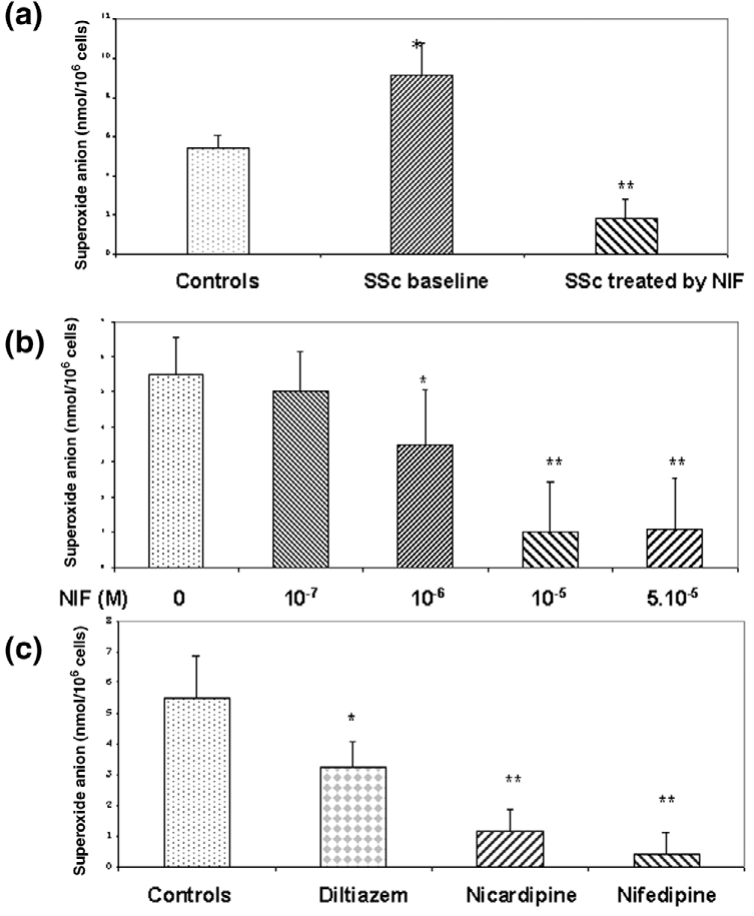
Superoxide anion production by monocytes after induction with phorbol myristate acetate (PMA; 100 nM) for 15 min. From healthy controls (*n *= 10), patients with systemic sclerosis (SSc) after disruption of calcium-channel blocker treatment (*n *= 12) (baseline) and patients with SSc after 14 days of treatment with 60 mg/day nifedipine (NIF) (*n *= 12) **(a)**, from healthy donors (*n *= 5) treated with or without various concentrations of nifedipine for 30 min before stimulation with PMA **(b) **and from healthy donors treated with or without calcium-channel blockers (10 µM) before stimulation with PMA **(c)**. *P < 0.05; **P < 0.01 relative to controls.

Monocytes from patients with SSc (*n *= 12) not treated by nifedipine produced a significantly greater amount of O_2_^•- ^than controls (*n *= 10) after stimulation *ex vivo *with the PKC activator PMA for 15 min (9.1 ± 1.7 versus 5.4 ± 0.7 nmol per 10^6 ^cells; *P *< 0.01; Fig. 1a). This difference was also observed when monocytes were stimulated with PMA for 10 min (7.6 ± 2.3 versus 4.3 ± 0.8 nmol per 10^6 ^cells; *P *< 0.01) but not when they were stimulated for 5 min (2.7 ± 0.8 versus 2.1 ± 0.4 nmol per 10^6 ^cells; not significant). These observations suggest that the biochemical modifications affecting monocytes from patients with SSc might not affect the kinetics of NADPH oxidase but rather its late regulatory mechanisms.

Monocytes from patients treated with nifedipine (60 mg/day) for 14 days produced a significantly smaller amount of O_2_^•- ^than monocytes from untreated patients with SSc after stimulation with PMA for 5 min (1.0 ± 0.7 versus 2.7 ± 0.8 nmol per 10^6 ^cells; *P *< 0.05), 10 min (1.6 ± 1.4 versus 7.6 ± 2.3 nmol per 10^6 ^cells; *P *< 0.001) and 15 min (1.8 ± 1.0 versus 9.1 ± 1.7 nmol per 10^6 ^cells; *P *< 0.001; Fig. 1a). Disease subsets, in particular the cutaneous subtype, did not influence baseline O_2_^•- ^production by monocytes or the decrease in O_2_^•- ^production with nifedipine treatment.

To determine whether monocytes were directly altered by nifedipine, monocytes from healthy donors were treated *in vitro *with nifedipine for 30 min before stimulation with PMA. Nifedipine induced a concentration-dependent inhibition of O_2_^•- ^production at concentrations of 10 and 50 µM (*n *= 5) (Fig. 1b). PMA induction was inhibited by 50% (IC_50_) by approximately 3–5 µM nifedipine. This inhibitory effect of nifedipine was not due to any cytotoxic effect, as determined by the trypan blue exclusion test (cell death less than 5%). The effects of nifedipine were next studied on the monocyte respiratory burst induced by an inflammatory agonist such as fMLP. However, fMLP induced very weak monocyte O_2_^•- ^production in standard conditions, so respiratory burst was measured with a fluorimetric assay. Treatment of monocytes for 30 min with 5 and 10 µM nifedipine significantly inhibited hydrogen peroxide production by 55 ± 7% and 90 ± 9%, respectively, relative to controls (*n *= 3).

Next we studied whether other calcium-channel blockers alter the O_2_^•- ^production of healthy monocytes (*n *= 5) (Fig. 1c). A strong and similar inhibitory effect was observed with nifedipine or nicardipine, but a less marked inhibitory effect was observed with diltiazem. To investigate whether the inhibitory effect of nifedipine was due to the scavenging of free radicals, O_2_^•- ^was generated by the xanthine/xanthine oxidase system in the presence of various concentrations of nifedipine. In these conditions, nifedipine did not affect O_2_^•- ^generation, ruling out a scavenger effect (data not shown).

### Protein phosphorylation

**Figure 2:**
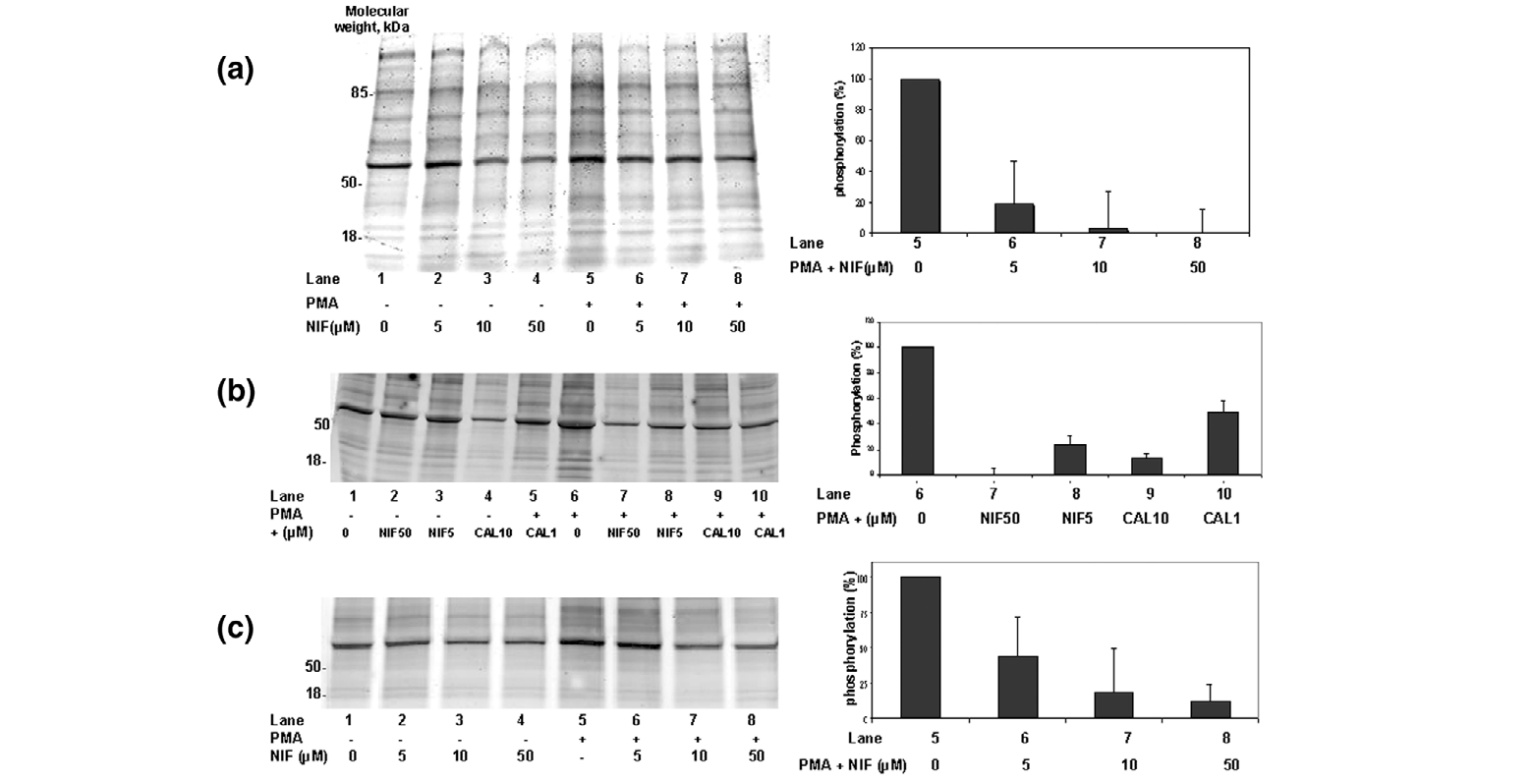
Effects of nifedipine (NIF) on phorbol myristate acetate (PMA)-induced protein phosphorylation in monocytes as detected with the fluorescent probe ProQ-Diamond. **(a, b) **Representative gels of phosphorylated protein from homogenates of monocytes from healthy donors, treated with the indicated concentrations of nifedipine (a, b) or calphostin (CAL) (b) for 30 min and then stimulated with 100 nM PMA for 10 min. Right panels show a representative densitometric analysis of 10 protein bands (molecular masses from 15 to 60 kDa) that were strongly phosphorylated in the presence of PMA. **(c) **Effects of nifedipine on the cytosolic fraction of control monocytes treated *in vitro *with the indicated concentrations of nifedipine for 30 min before stimulation of protein kinase C with a mixture of calcium and diacylglycerol. Results are the net phosphorylation induced by PMA, expressed relative to the PMA lane (taken as 100%).

The production of O_2_^•- ^by phagocytes is dependent on the activation of PKC, a family of isoenzymes that phosphorylate various proteins including some components of the NADPH oxidase. Because PMA directly activates various PKC isoforms, we studied the effect of nifedipine on PKC-dependent protein phosphorylation in healthy monocytes. Phosphorylated proteins were directly detected in polyacrylamide gels by using Pro-Q-Diamond, a fluorescent dye that binds to phosphate groups on proteins. In the absence of PMA, nifedipine inhibited the basal phosphorylation of various proteins in monocytes (Fig. 2a). In monocytes not treated with nifedipine, stimulation with PMA markedly increased the phosphorylation state of various proteins and nifedipine strongly decreased the PMA-induced phosphorylation of proteins. Densitometric analysis of 10 major phosphorylated protein bands with molecular masses of between 60 and 15 kDa showed that nifedipine inhibited the net protein phosphorylation induced by PMA in a concentration-dependent manner. The inhibition of phosphorylation was also investigated in this cell system under the same conditions with the classical PKC inhibitor calphostin; the inhibition of protein phosphorylation had a quite similar profile (Fig. 2b) with closed results on densitometric analysis.

The inhibitory effects of nifedipine were also demonstrated in a cell-free system in which the activity of PKCs present in the cytosolic fraction of resting monocytes was directly stimulated in the presence of a mixture of calcium and diacylglycerol. In these conditions, treatment of the cytosolic fraction with nifedipine abolished PKC-dependent protein phosphorylation (Fig. 2c).

### PKC activity *in vitro*

**Figure 3:**
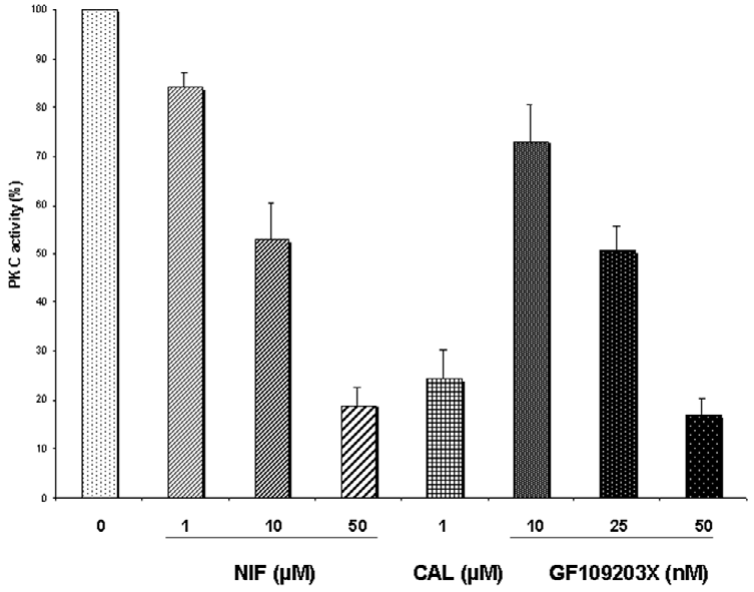
Nifedipine (NIF), like the classical protein kinase C (PKC) inhibitors calphostin (CAL) or GF109203X, inhibited the PKC activity of recombinant PKC as evaluated by an enzyme-linked immunosorbent assay method (*n *= 3). Results are the net phosphorylation induced by phorbol myristate acetate (PMA), expressed relative to the PMA lane (taken as 100%).

To determine whether PKC is a direct target of nifedipine, we investigated kinase activity *in vitro *with a biologically active PKC recombinant that was pretreated with or without various concentrations of nifedipine or with other classical PKC inhibitors such as calphostin or GF109203X [16]. Nifedipine (1, 10 and 50 µM) dose-dependently inhibited PKC activity with an inhibition reaching about 80%. Similar inhibition was observed with calphostin and GF109203X (*n *= 3; Fig. 3). These results strongly suggest that nifedipine might directly inhibit PKC.

## Discussion

In this report we show that, first, *ex vivo *monocytes from patients with SSc not treated with calcium-channel blockers produce more O_2_^•- ^than control monocytes; second, nifedipine (60 mg/day) strongly inhibits the ability of monocytes to produce O_2_^•- ^*ex vivo*; and third, nifedipine inhibits O_2_^•- ^production by healthy monocytes *in vitro*, a property associated with the inhibition of PKC-dependent protein phosphorylation and PKC activity.

Since the proposal that free radicals have a function in SSc [17], several reports have provided evidence that free radicals are involved in the pathogenesis of this complex disease [1,2,7,18]. Vasospasm and ischaemia–reperfusion are known to generate free radicals, but other factors such as the inflammatory process might also be involved [1]. It has been suggested that resting and PMA-stimulated monocytes produce more O_2_^•- ^than controls [7]. This is consistent with our observation that circulating monocytes from patients with SSc stimulated *ex vivo *by PMA release about twice as much O_2_^•- ^as controls, which is similar to previous findings [7]. This PMA-dependent increase in O_2_^•- ^suggests that circulating monocytes are in a primed state, which is consistent with their prior exposure to a substimulatory dose of agonists such as cytokines or immune complexes [19]. These data show that free radicals are overproduced in SSc and that this is due at least partly to monocytes. The mechanism of monocyte activation remains unknown. However, ischaemic insult does not seem sufficient to explain this phenomenon, which is associated with NADPH oxidase activation and might involve cytokines [7].

The main finding of this study is that the treatment of patients with SSc with nifedipine (60 mg/day) strongly decreases the production of O_2_^•- ^by monocytes and leads to the disappearance of the monocyte priming state. PMA was chosen because it mimics the activation of NADPH oxidase by PKC, a mechanism also induced by most of the physiological activators of monocytes. Mononuclear cells are thought to be important in SSc [3-5] and in oxidative stress because they release various mediators leading to tissue injury. Unlike monocytes, polymorphonuclear neutrophils do not seem to be activated in SSc [7]. The results reported here are consistent with our previous data showing that dihydropyridine-type calcium-channel blockers can reduce plasma markers of oxidative stress [10]. Together with data showing a concomitant improvement of endothelial injury [11], these results suggest that calcium-channel blockers, especially the dihydropyridine type, are not only of major importance for the treatment of Raynaud's phenomenon and vasospastic propensity [20,21] but should also be regarded as essential drugs that limit monocyte activation and oxidative stress.

Several reports have demonstrated the anti-oxidant properties of calcium-channel blockers. In hypertensive rats, dihydropyridine calcium-channel blockers decrease lipoprotein oxidation and prolong survival independently of modifications of blood pressure [22]. In hypertensive patients, treatment with nifedipine decreases lipoperoxide and isoprostane concentrations and increases the plasma antioxidant capacity while increasing endothelium-dependent vasodilation by restoring nitric oxide bioavailability [23]. At the cellular level, nifedipine can prevent ischaemia-induced endothelial permeability mediated primarily by the inhibition of PKC-a [24]. A study with activated human and rabbit neutrophils [25] and another study in vascular smooth muscle cells [26] suggested that PKC can be inhibited by nicardipine. Our results are consistent with these findings and further emphasise the ability of nifedipine to inhibit PKC activity *in vitro*, as shown by the strong decrease in PMA-induced protein phosphorylation in monocytes and in a cell-free system and the inhibition of a recombinant isoform of PKC. Analysis of the protein phosphorylation pattern showed that nifedipine also inhibits basal phosphorylation of proteins, indicating that other protein kinases might be altered. The molecular mechanism by which nifedipine inhibits PKC cannot be derived from our results and will require further experiments. However, nifedipine inhibits both basal and PMA-induced protein phosphorylation as well as PKC activity, which suggests that nifedipine might interact with both the regulatory and catalytic domains of PKC.

The inhibition of PKC-dependent protein phosphorylation might be part of the molecular mechanism responsible for the loss of the primed state of monocytes from patients with SSc treated with nifedipine. This is emphasised by the fact that IC_50 _values were above 3–5 µM nifedipine for PMA-induced O_2_^•- ^production *in vitro *(Fig. 1b) and also for PKC-dependent phosphorylation (Fig. 2a). However, this hypothesis must be tested with monocytes from patients with SSc.

Nifedipine (10 and 50 µM) significantly inhibited O_2_^•- ^release by activated monocytes *in vitro*; this is consistent with results previously obtained with human neutrophils [25]. The IC_50 _for calcium channels antagonist activity of nifedipine is close to 100 pM [27] and the clinical plasma concentration of nifedipine is close to 0.2 µM [28]. We used higher nifedipine concentrations for experiments *in vitro*; however, nifedipine can accumulate in membrane-bounded structures, resulting in higher localised concentrations [29]. Thus, our data with nifedipine and other calcium-channel blockers *in vitro *could explain the results obtained with monocytes from patients with SSc treated with nifedipine.

We found that diltiazem had a weaker effect on O_2_^•- ^production by activated monocytes than other calcium-channel blockers evaluated; this was previously reported using human neutrophils [30] and suggests that dihydropyridine-type calcium-channel blockers are the strongest inhibitors of O_2_^•- ^production.

Most of the classical properties of nifedipine are exerted through the modulation of L-type voltage-gated calcium channels on smooth muscle cells. Such channels are also present on monocytes; we suspect that the molecular mechanism highlighted is independent of these channels, but this will require further investigations. Calcium channel-independent properties have previously been reported and because these are not represented in endothelial cells they cannot account for these cellular effects [31]. It was suggested that these drugs act as scavengers; however, our data, together with those on human polymorphonuclear neutrophils [24], do not support this hypothesis.

## Conclusion

Monocytes from patients with SSc adopt a priming state resulting in increased *ex vivo *PKC-dependent production of O_2_^•- ^relative to healthy monocytes. Treatment of patients with SSc or of healthy monocytes *in vitro *markedly decreased the ability of monocytes to generate O_2_^•- ^. Biochemical analysis of protein phosphorylation in monocytes and cell-free systems suggested that nifedipine directly inhibits PKC activity. These results demonstrate the potential anti-oxidant effects of this drug, which might have important clinical implications for SSc and other oxidative stress-associated diseases.

## Abbreviations

DTCCA = dihydropyridine-type calcium-channel antagonists; fMLP = formyl-Met-Leu-Phe; IC_50 _= concentration for 50% inhibition; PKC = protein kinase C; PMA = phorbol myristate acetate; SSc = systemic sclerosis.

## Competing interests

The author(s) declare that they have no competing interests.

## Authors' contributions

YA, DB and AP devised the study. YA, HL and AP performed all the experiments. OGE and AK assisted in the writing of the report. All authors read and approved the final manuscript.

## Figures and Tables

**Table 1:** Clinical and biological characteristics of patients with systemic sclerosis (SSc)

Characteristic	Limited SSc (*n *= 6)	Diffuse SSc (*n *= 6)
Raynaud's phenomenon, *n*	6	6
Current digital vascular ulceration, *n*	0	0
Lung fibrosis (computed tomography scan), *n*	2	4
Forced vital capacity <75%, *n*	0	4
DLCOc/haemoglobin <80%, *n*	3	3
Pulmonary hypertension (sPAP >40 mmHg), *n*	0	0
Positive for anti-topoisomerase I antibodies, *n*	0	4
Positive for anti-centromere antibodies, *n*	3	0
Creatinine, µmol/l (range)	73.4 ± 12.5^a ^(56–81)	76.4 ± 13.5^a ^(60–90)
ESR, mm/h	13.6 ± 11^a^	18.6 ± 15^a^
C-reactive protein concentration, mg/l	7.6 ± 6.1^a^	8.8 ± 7.4^a^
Low dose of prednisone (ongoing treatment), *n *(mean mg/day)	2 (7)	4 (7.5)
Angiotensin-converting enzyme inhibitors, *n*	0	3
D-Penicillamine, *n*	0	2
Omeprazole, *n*	6	6

^a^Mean ± SD.

DLCOc, carbon monoxide diffusing capacity of the lung corrected for haemoglobin; ESR, erythrocyte sedimentation rate; sPAP, systolic pulmonary arterial pressure.
